# Rapid Colonisation of Plastic Surfaces by Marine *Alcanivorax* Bacteria Is Flagellum‐Dependent and Influenced by Polymer Type and Photo‐Weathering State

**DOI:** 10.1111/1462-2920.70102

**Published:** 2025-05-02

**Authors:** Keren Davidov, Sheli Itzahri, Aiswarya Kartha, Gilad Orr, Ziv Lang, Shiri Navon‐Venezia, Matan Oren

**Affiliations:** ^1^ Molecular Biology Department Ariel University Ariel Israel; ^2^ Physics Department, Crystal Physics Laboratory Ariel University Ariel Israel; ^3^ The Sheldon Adelson School of Medicine Ariel University Ariel Israel

**Keywords:** *Alcanivorax*, bacterial colonisation, bacterial motility, bacterial surface adhesion, flagellum, plastic photo‐weathering, plastisphere, polymyxin B sulfate

## Abstract

Marine plastic debris provides stable surfaces for microbial colonisation, forming a unique ecosystem known as the plastisphere. Among the early colonisers are *Alcanivorax* bacteria, hydrocarbon degraders commonly found in oil‐polluted seawater and on marine plastic surfaces. This study examined factors influencing the adhesion and colonisation dynamics of six *Alcanivorax* species. Flagellated species— *A. balearicus* , *A. dieselolei* and *A. xenomutans*—rapidly colonised plastics, particularly polyethylene and polypropylene, while non‐flagellated species did not. Notably, plastic photo‐weathering treatments led to the elongation of *A. dieselolei* cells, secretion of extracellular polymeric substance in some cases, and increased colonisation on UVB‐treated polyethylene terephthalate. These changes may be linked to the reduced plastic surface hydrophobicity recorded following photo‐weathering. To confirm the role of flagella in *Alcanivorax* adhesion, we disrupted flagellar activity using sub‐concentrations of polymyxin B sulfate, resulting in inhibition of swarming motility and complete disruption of colonisation. These results contribute to our understanding of the interactions between hydrocarbon‐degrading *Alcanivorax* bacteria and their plastic substrate, which in turn contributes to the understanding of the ecological impact of plastic pollution in marine environments.

## Graphical abstract

Marine hydrocarbon‐degrading *Alcanivorax* bacteria are abundant on plastic debris in the Mediterranean Sea. Our study explores the role of flagella in the colonisation of plastic surfaces by six *Alcanivorax* species and examines how plastic polymer type and photo‐weathering state influence bacterial adhesion and colonisation dynamics.

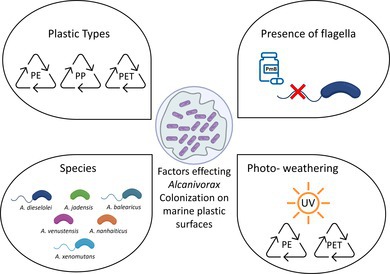

## 1 Introduction

Plastic pollution is one of the most pressing environmental issues of the 21st century, with over 400 million tons of plastic produced annually (PlasticsEurope 2023) and a significant portion entering marine ecosystems (Jambeck et al. 2015). Unlike natural organic materials, plastics persist in the environment for decades or even centuries, leading to the accumulation of plastic debris in oceans, rivers and coastal areas. In aquatic environments, plastic surfaces are rapidly colonised by microorganisms, initiating the formation of biofilms. The process begins with the reversible attachment of microbial cells to the surface, facilitated by organelles such as pili, flagella and/or adhesive membrane proteins (Berne et al. 2018). Once attached, the microbes secrete extracellular polymeric substances (EPS), securing their adhesion and transitioning to an irreversible state. Over time, the EPS becomes thicker, and a mature biofilm is formed (Zhai et al. 2023). The mature plastic‐attached biofilms host diverse microbial communities, including bacteria, fungi and algae (Davidov et al. 2020), displaying intricate inter‐taxa interactions (e.g., Itzahri et al. 2023). Together, the plastic surfaces and their associated microbial communities form a distinct ecosystem known as the plastisphere (Amaral‐Zettler et al. 2020).

The adhesion of a bacterial cell to a surface is determined by the sum of attractive and repulsive forces (Ren et al. 2018). Most pristine plastic polymers are highly hydrophobic. However, in the marine environment, they undergo physical and chemical weathering processes that reduce their hydrophobicity. Several factors contribute to plastic weathering in the ocean, including exposure to light and heat radiation, as well as the mechanical forces exerted by waves and currents (Andrady 2022). Photo‐oxidation of the polymeric chains is a major driver of plastic weathering, with ultraviolet B (UVB) radiation (280–315 nm) being particularly effective (Andrady et al. 2022). In addition to reducing hydrophobicity, plastic weathering processes fragment the material into micro‐ and nano‐plastics, lower the molecular weight of the polymers (Andrady 2022), and increase the surface area, thereby enhancing the susceptibility of plastic surfaces to microbial colonisation.

Among the microbial inhabitants of the plastisphere, bacteria of the genus *Alcanivorax* (Class Gammaproteobacteria) have generated significant attention. The hydrocarbonoclastic genus *Alcanivorax* includes species capable of degrading and metabolising medium‐ to long‐chain alkanes (Wang and Shao 2014; Sabirova et al. 2011; Rojo 2009) and thus thrives in oil‐contaminated seawater (Kasai et al. 2002; Hara et al. 2003; Cappello et al. 2007; Harayama et al. 2004; de Queiroz et al. 2024; Yakimov et al. 2019). *Alcanivorax* bacteria were also found in multiple studies on marine plastic debris (Delacuvellerie et al. 2019; Zadjelovic et al. 2020a). We have recently shown that *Alcanivorax* is a highly abundant genus on floating plastic surfaces in the Eastern Mediterranean Sea, specifically on polyolefins, including polyethylene (PE) and polypropylene (PP) (Davidov et al. 2020; Marsay et al. 2023, 2022). In an in situ experiment, we showed that *Alcanivorax* bacteria rapidly colonised these polymers, emerging as the dominant genus within the first few days and maintaining dominance for up to a month after surface immersion in seawater (Davidov et al. 2025). Furthermore, evidence suggests that certain *Alcanivorax* strains can degrade and metabolise long‐chain plastic polymers, including low‐density polyethylene (Delacuvellerie et al. 2019; Zadjelovic et al. 2022; Khandare et al. 2021), PP (Koike et al. 2023) and Polystyrene (PS) (Liu et al. 2023).

To promote the biodegradation of plastics in aquatic environments, initial adhesion mechanisms of microorganisms to polymer surfaces are essential. Using forward genetics, Wang and Shao identified an alkane chemotaxis complex in *Alcanivorax dieselolei* , which is hypothesized to guide the bacterium toward higher concentrations of linear alkane molecules in marine settings (Wang and Shao 2014). However, it remains unclear whether this chemotaxis mechanism also facilitates *Alcanivorax* movement toward solid plastic surfaces in the marine environment. Moreover, the mechanisms underlying the irreversible adhesion of *Alcanivorax* to plastic surfaces, as well as the influence of surface properties on this process, have yet to be elucidated.

In this study, we investigated the ability of six *Alcanivorax* species to adhere and colonise common plastic polymers such as polyolefins (PE and PP) in comparison to Polyethylene Terephthalate (PET). We further evaluated the effect of plastic photo‐weathering on *A. dieselolei* colonisation. Finally, we tested the hypothesis that the presence of flagella in the fast plastic‐colonising strains is essential for their adhesion to the plastic surfaces.

## 2 Experimental Procedures

### 2.1 *Alcanivorax* Strains and Culturing Conditions

*Alcanivorax jadensis* (DSM 12178), *Alcanivorax venustensis* (DSM 13974), *Alcanivorax dieselolei* (DSM 16502) and *Alcanivorax balearicus* (DSM 23776) were purchased from the DSMZ collection (Germany), and *Alcanivorax xenomutans* (KCTC 23751) and *Alcanivorax nanhaiticus* (KCTC 52137) were purchased from the KCTC collection (Korea). Before each experiment, bacteria were inoculated from the stock onto MB (Marine Broth 2216, Sigma) agar plates at 28°C until visual colonies were formed. Single colonies were picked to establish MB liquid cultures. Logarithmic‐phase bacteria were used for different experiments.

### 2.2 *Alcanivorax* Colonisation Experiments on Plastic Surfaces

For the colonisation experiments, 0.2–0.6 mm thick sheets of three common plastic polymers were used: low‐density polyethylene (LDPE, IPETHENE 323, Carmel olefins, Israel), polypropylene (PP, CAPILENE E 50 E, Carmel olefins, Israel) and polyethylene terephthalate (PET, SKYPET BR, ResMarts, USA). The experiments were performed in carbon‐free Bushnell‐Haas basal mineral medium (BH, 0.2 g MgSO_4_, 0.02 g CaCl_2_, 1 g KH_2_PO_4_, 1 g K_2_HPO_4_, 1 g NH_4_NO_3_, 0.05 g FeCl_3_, pH = 7.0) supplemented with 30 g/L NaCl and 1 mL/L Trace element solution (Per 100 mL: 2.2 g ZnSO_4_·7H_2_O, 1.1 g H_3_BO_3_, 0.5 g MnCl_2_·4H_2_O, 0.5 g FeSO_4_·7H_2_O, 0.16 g CoCl_2_·6H_2_O, 0.16 g CuSO_4_·5H_2_O, 0.11 g (NH_4_)_6_MO_7_O_24_·4H_2_O and 6 g Na_4_EDTA·4H_2_O in deionised water, pH = 7.0) according to (Zadjelovic et al. 2020b). Each experimental setup contained mixed disks of three repeats of each LDPE, PP and PET polymers, 0.5 mm in diameter, that were tied 5 mm apart to a 0.4 mm fishing line. The plastic disks were sterilised using 70% ethanol and rinsed with sterile distilled water before the experiment. Sterile glass coverslips (0.1 mm thick) served as reference surfaces in the *A. dieselolei* colonisation experiment. The sterile surfaces were immersed in 50 mL flasks with 30 mL of BH medium with 1 mL of logarithmic‐phase *Alcanivorax* culture (OD 0.1 at 600 nm). The cultures were incubated at 28°C with shaking (100 rpm) for 24 to 168 h, depending on the experiment.

### 2.3 Quantification of *Alcanivorax* Cells on the Surfaces

Crystal Violet staining was used to estimate the quantity of *Alcanivorax* bacteria on the surfaces, according to (Stepanović et al. 2007). After incubation, surfaces were pulled out, washed with sterile filtered (0.22 μm) artificial seawater (FASW, 40 ppt, Red Sea salt, Red Sea Inc.) and air‐dried for 45 min. For quantification of colonised *Alcanivorax* biomass, the surfaces were stained with 1% Crystal Violet (V5265, Sigma) for 30 min at 150 rpm, washed, air‐dried and photographed. The crystal violet stain was eluted from the surfaces using 100% ethanol for 15 min on an orbital shaker at 150 rpm. The concentration of crystal violet elutions was measured using a microplate reader at a wavelength of 595 nm. All measurements were performed in triplicate. Surfaces that were not incubated with bacteria were stained as well and used as blanks. To normalise the glass surface results, OD values were divided by the surface area in cm^2^ according to the cylinder surface equation: *2πr* ^*2*^ *+ 2πrh* (*r*‐ radius, *h*‐ height).

### 2.4 *Alcanivorax* Phylogenetic Tree

The phylogenetic relationships among selected bacterial genomes were created using the Bacterial Genome Tree tool available at the BV‐BRC platform (https://www.bv‐brc.org/) (Olson et al. 2023). The analysis employed the Codon Tree method, focusing on single‐copy BV‐BRC PGFams (Protein Families) from the selected genomes. A phylogenetic tree was constructed based on alignments of both protein sequences and coding DNA sequences from 500 single‐copy genes. The resulting tree was constructed using RAxML (Randomised Axelerated Maximum Likelihood) to ensure robust maximum‐likelihood estimation and accurate representation of evolutionary relationships.

### 2.5 Plastic Weathering

PE and PET plastic sheets were subjected to accelerated weathering using two types of light radiation: 1. Full‐cell Xenon sunlight spectrum, using a Q‐SUN XE‐3 Xenon Test Chamber (Q‐Lab) at an intensity of 60 W/m^2^, at 60°C with water spray every 90 min for 3 months. 2. UVB radiation at a wavelength of 280–315 nm at an intensity of 1.2 W/m^2^/nm using a QUV accelerated weather tester (Q‐Lab) for 7 days.

### 2.6 Fourier‐Transform Infrared Spectroscopy (FTIR)

FTIR spectral analyses were performed to identify chemical changes in functional groups and the polymer backbone, comparing weathered polymers to pristine samples. The FTIR was carried out using a JACSOFT/IR‐6800 spectrometer. Spectra were obtained in the wavelength range of 600–4000 cm^−1^ and the resolution was fixed at 2 cm^−1^. A small plastic fragment of each sample was pressed under the K/Br crystal apparatus for measurements. The absorbance spectra were visualised with OpenSpecy, an open‐source library (Cowger et al. 2021) with baseline correction. For the determination of PE degradation, we calculated carbonyl (C=O) and vinyl (C=C) indexes using the following formulas:

$$\text{Carbonyl index}=A_{1715}/A_{1478}$$

$$\text{Vinyl index}=A_{910}/A_{1478}$$

where *A*_1715_ and *A*_910_ represent the absorbance at 1715 cm^−1^ (carbonyl) and 910 cm^−1^ (vinyl), respectively, and *A*_1478_ corresponds to the reference peak for PE.

### 2.7 Sessile Drop Test

Weathering introduces changes in surface structure and bond structure of the polymers at the surface. The sessile drop method was applied to test the changes due to the weathering. The setup consisted of a Sony IMX477 high‐resolution image sensor (12.3MP) mounted via a C‐mount adapter to a camera microscope with variable magnification. Opposite to the camera microscope was an x‐y‐z micrometre translation stage with a tilting stage and diffused backlight illumination. This allowed for adjusting the drop's imaging distance, providing a sharp and levelled image. The samples were attached using double‐sided tape to microscope slides, which were placed on the stage opposite to the camera microscope. A 1 μL highly purified distilled water drop was presented to the surface of the sample using a micro‐pipette, and an image of the drop was taken for analysis after adjusting its distance from the camera microscope.

The contact angle was estimated by using a method based on the Young–Laplace (Figure S1) (Stalder et al. 2010).

### 2.8 Scanning Electron Microscopy (SEM)

To observe the effect of photo weathering on the plastic surfaces by SEM, clean pieces of Xenon‐treated, UVB‐treated and pristine polymers of PE and PET sheets were used without any further processing. Surfaces containing bacteria or bacterial biofilm were first fixed with 2%–2.5% glutaraldehyde for 1–3 h, washed three times with distilled water for 5 min and dehydrated in a graded ethanol series for 10 min each in 10%, 20%, 30%, 40%, 50%, 70%, 85% and 95% ethanol, followed by 3 × 15 min in 100% ethanol. Subsequently, samples were dried using a critical dry point (Quorum K850 Quorum Technologies Ltd. Lewes, UK). Finally, samples were sputter‐coated with 10 nm of platinum/gold (Quorum Q150T ES) and then visualised and imaged on an Ultra‐High‐Resolution Maia 3 FE‐SEM (Tescan) in a range of 3–7 kV voltage.

### 2.9 Transmission Electron Microscopy (TEM)

To visualise bacterial morphology and characterise flagella, we used counterstaining (Stalder et al. 2010). A fresh bacterial pellet was washed and fixed overnight on an orbital shaker in paraformaldehyde 3% glutaraldehyde 0.35% in 0.1 M sodium cacodylate buffer (pH 7.4, 15,949, EMS). The samples were then stored at 4°C until further use. Before visualisation, 200 mesh carbon‐coated copper grids were glow discharged with the Emi Tech K100 machine, and then 5 l of the sample was loaded onto the grid. After 1 min, the sample was blotted, excess material was removed, then 5 l of 1% uranyl acetate was loaded for 30 s, blotted and washed with Double‐Distilled (DD) water, then air dried. The sample was then inspected with a JEM 1400 microscope (JEOL) with an acceleration voltage of 120 kV. Images were taken using a Digital Micrograph with a Militician Camera model 794 (Gatan). TEM imaging was performed at the Institute of Nanotechnology and Advanced Materials, Bar‐Ilan University (Israel).

### 2.10 BLAST Ring Image Generator (BRIG) of *Alcanivorax* Flagellum Genes

The genome sequences of the six *Alcanivorax* species were compared to the *Alcanivorax* sp. 24 flagellum locus (Zadjelovic et al. 2020a). The comparison was made with the BRIG (version 0.95‐dev.0004) and GToTree (version 1.7.06) software.

### 2.11 Soft Agar Swarming Motility Assay

To assess the bacterial swarming motility, we performed a soft agar motility assay (Kearns 2010). Bacterial cultures were grown to the logarithmic phase and centrifuged for 8 min at 6000 rpm. The cells were washed twice with autoclaved, filtered (0.22 μm) artificial seawater (40 ppt, Red Sea salt, Red Sea Inc.) and resuspended to an OD of 0.3. A 3 μL droplet of the culture was placed in the centre of the 0.5% (W/V) MB agar plate and incubated for 48 h at 28°C. The diameters of the colonies were measured before and after incubation using a binocular microscope (SMZ25, Nikon). Images were captured and analysed using NIS‐Elements software (Nikon).

### 2.12 Determination of the Minimum Sub‐Inhibitory Concentration of Polymyxin B Sulfate Salt

The sub‐inhibitory concentration of PmB (P4932, Sigma) was defined as a concentration that does not impact bacterial viability. To find this concentration, *A. dieselolei* , *A. balearicus* and *A. xenomutans* were grown in MB medium supplemented with 0.1, 0.25 and 0.5 μg/mL of PmB. Bacterial growth was monitored over 72 h by measuring absorbance at 600 nm using a 96‐well plate reader (Tecan). The validation of flagellum disturbance by the chosen PmB sub‐dose (0.1 μg/mL) was performed by TEM imaging and soft agar swarming assay as described above.

### 2.13 Statistical Analysis

Statistical analyses were performed using GraphPad Prism software (version V8.0.2). Data normality was assessed using the Shapiro–Wilk test. For normally distributed data, statistical comparisons were made using either a one‐way or two‐way analysis of variance (ANOVA), depending on the experimental design. For significant ANOVA results, post hoc tests (e.g., Tukey's or Bonferroni) were applied to determine specific differences between groups while controlling for multiple comparisons. Statistical significance is represented graphically as * for *p* < 0.05, ** for *p* < 0.01 and *** for *p* < 0.001.

## 3 Results

### 3.1 The Effect of Plastic Polymer Type on the Colonisation of Different *Alcanivorax* Species

**FIGURE 1:**
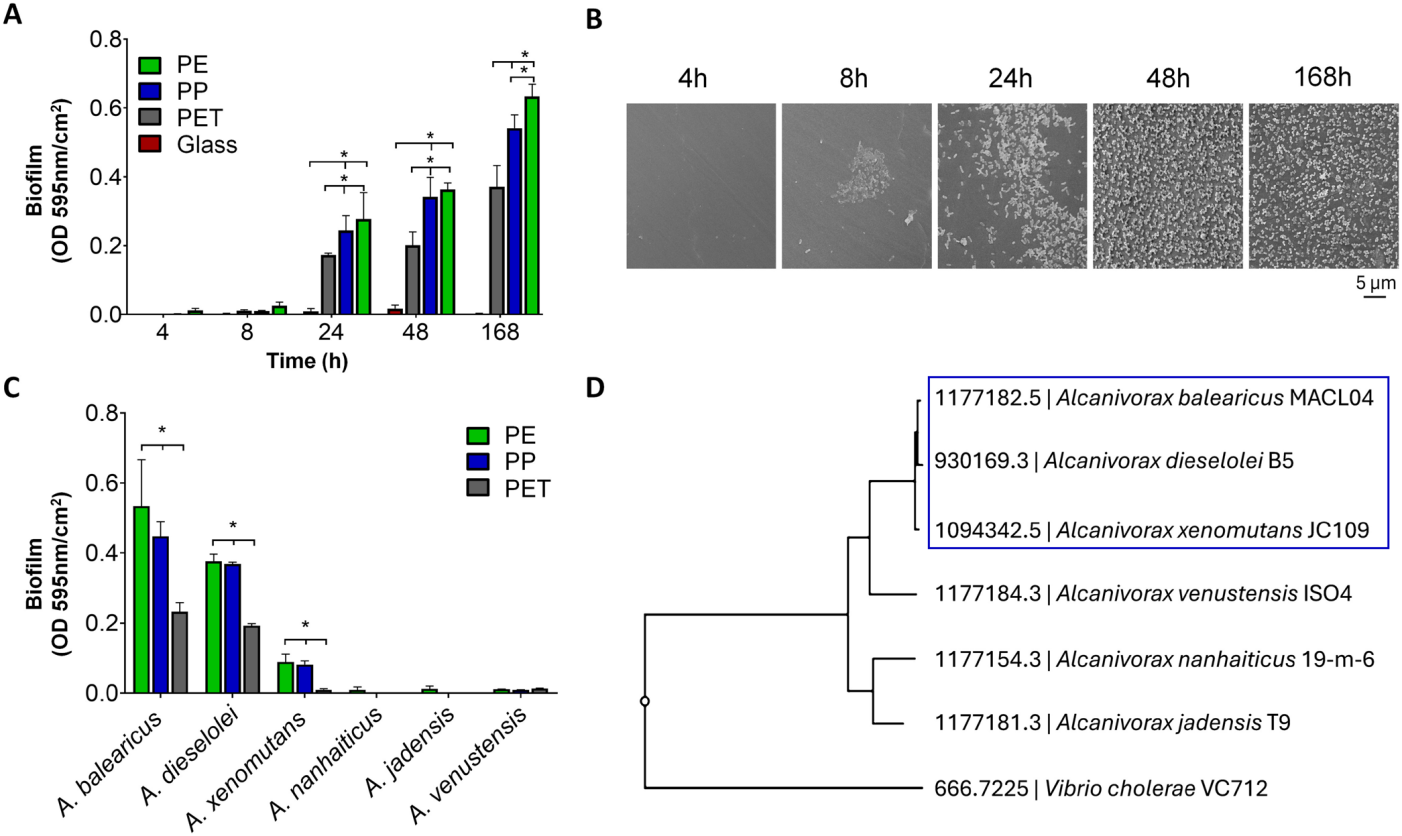
Colonisation patterns of *Alcanivorax* species on pristine PE, PP and PET surfaces. (A) *A. dieselolei* colonisation on PE, PP, PET and glass over 4 to 168 h in BH media (Crystal Violet staining). (B) SEM images of plastic‐colonising *A. dieselolei* cells on PE surface over 4 to 168‐h period. (C) Colonisation of six *Alcanivorax* species on pristine PE, PP and PET surfaces after 48 h of incubation in BH media (Crystal Violet staining). (D) Phylogenetic tree of *Alcanivorax* species according to the codon tree method based on sequences of 500 single‐copy genes (https://www.bv‐brc.org/). Rapid coloniser species are marked. Statistical significance was determined by Two‐way ANOVA: *p < 0.05.

To investigate *Alcanivorax* colonisation kinetics on the different polymer types, we first estimated the surface bacterial cell coverage on PE, PP, PET and glass coverslips after 4, 8, 24, 48 and 168 h (7 days) of incubation in carbon‐free BH medium with *A. dieselolei* bacteria. After 24 h, bacterial colonisation was observable by Crystal Violet staining on all plastic surfaces. The colonisation was significantly higher on polyolefin polymers compared to PET throughout the entire experiment, with Crystal Violet optical density (OD) at 595 nm per cm^2^ mean values of 0.36 ± 0.1 and 0.34 ± 0.05 for PE and PP respectively, compared to only 0.2 ± 0.03 for PET at the 48‐h time point. No Crystal Violet staining was detected on glass throughout the entire experiment, suggesting no bacterial colonisation on this type of surface within the tested timeframe (Figure 1A).

SEM imaging showed patches of *A. dieselolei* bacteria on pristine PE surfaces as early as 8 h from the bacterial exposure to the surface (Figure 1B). The bacterial colonisation patches significantly expanded after 24 h of incubation, reaching almost confluent surface coverage 48 h post‐incubation. While the overall coverage remained the same at the 168‐h time point, bacterial cells seemed to adhere to each other in small clusters of 2–10 bacteria each, without forming any visible extracellular polymeric substance (EPS). Based on these findings, we conducted all subsequent colonisation assays at the 48‐h incubation time point, when full bacterial coverage can potentially be observed.

The 48‐h incubation test for the six *Alcanivorax* species resulted in detectable plastic colonisation by three species: *A. balearicus* , *A. dieselolei* and *A*. *xenomutans*, whereas the other three: *A. nanhaiticus* , *A. jadensis* and *A. venustensis*, did not attach to any of the surfaces at the 48‐h time point (Figure 1C, Figure S2). Interestingly, based on Crystal Violet staining, all plastic‐colonising species showed significantly higher cell coverage on polyolefin surfaces than on PET. The Crystal Violet average OD values were 2.1 times higher on polyolefin than on PET when incubated with *A. balearicus* , 1.9 times higher when incubated with *A. dieselolei* , and 9 times higher when incubated with *A. xenomutans* (Figure 1C).

The differences in colonisation patterns among the six *Alcanivorax* species correlated with the phylogenetic groupings based on a codon tree analysis of 500 single‐copy genes. The analysis resulted in the clustering of the three colonising species within the same clade (Figure 1D), suggesting a genetic basis for rapid plastic colonisation.

### 3.2 The Effect of Plastic Polymer Weathering on the Colonisation of *Alcanivorax dieselolei*

**FIGURE 2:**
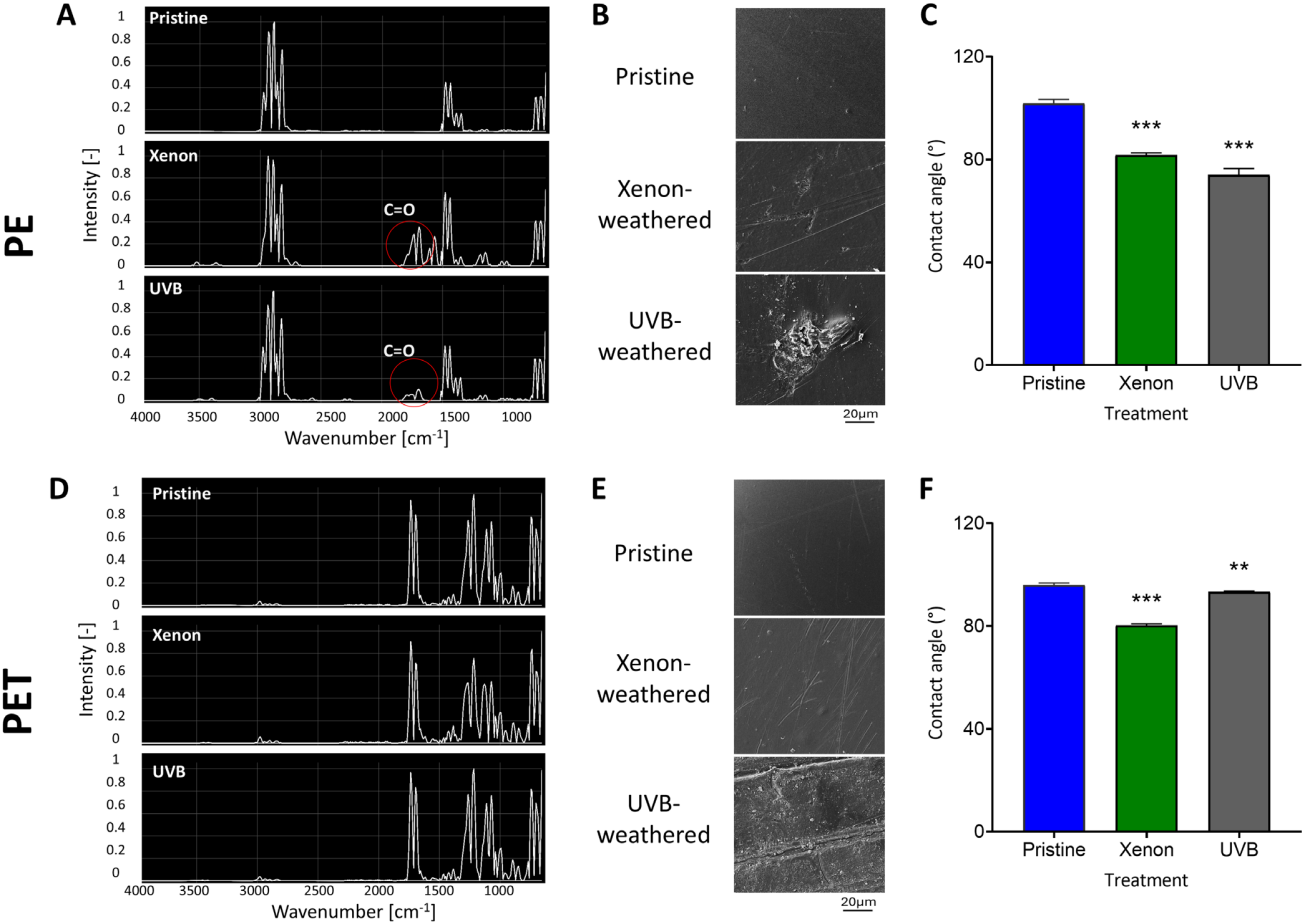
Physical and chemical changes to PE and PET surfaces after exposure to artificial daylight (Xenon) and ultraviolet radiation (UVB light source). (A, D) FTIR spectra of PE (A) and PET (D) surfaces before and after photo‐weathering treatment. The red circle indicates the post‐treatment appearance of a carbonyl group (C=O) at ~1740 cm^−1^ in the PE surfaces. No apparent IR absorbance difference was observed in the weathered PET surfaces. (B, E) SEM pictures of the surfaces showing scratches and pits on the photo‐weathered surfaces. (C, F) Hydrophobicity of the plastic surfaces. Contact angle measurement of 1 μL water droplet on pristine and weathered plastic surfaces. The plastic surface contact angle was significantly decreased following photo‐weathering. **p value < 0.01, ***p value < 0.001 (one‐way ANOVA).

To investigate how plastic photo‐weathering affects *Alcanivorax* colonisation, we exposed PE and PET sheets to accelerated radiation of full sunlight spectrum (intensity of 60 W/m^2^, at 60°C with water spray every 90 min for three months) and ultraviolet B (260 nm, at an intensity of 1.2 W/m^2^/nm for 7 days). Increased brittleness was observed in both polymer surfaces. UVB exposure induced a yellow tint in PET. The FTIR analysis of the weathered PE resulted in the appearance of a new absorbance peak at the wavelength of 1715 cm^−1^ following both treatments, indicating the formation of carbonyl groups (C=O). The carbonyl index was 1.57 for the Xenon‐treated PE and 1.3 for the UVB‐treated PE versus 0 for the pristine polymer indicating photo‐oxidation of the polymer (Figure 2A). The Vinyl index values increased from 0.041 for the pristine PE to 0.62 for the Xenon‐treated PE and 0.89 for the UV‐B‐treated PE indicating degradation or structural alterations in the polymer. We could not detect any photo‐weathering‐induced absorbance spectrum changes in the PET (Figure 2D). Nevertheless, SEM imaging revealed scratches and micro‐holes in both PE and PET surfaces (Figure 2B,E, Figure S3).

Xenon and UVB weathering reduced surface hydrophobicity in both polymers (Figure 2C,F). Pristine PE showed the highest hydrophobicity rate with an average contact angle of 101.7° compared with pristine PET with an average contact angle of only 95.9°. Xenon and UVB treatments significantly reduced PE hydrophobicity, showing 19.7% and 27.3% reductions in the droplet contact angle values, respectively. Weathered PET samples showed a milder reduction (yet significant) in surface hydrophobicity as deduced from the decrease of 2.6% for Xenon treatment and 16.2% for UVB treatment in the droplet contact angle values.

**FIGURE 3:**
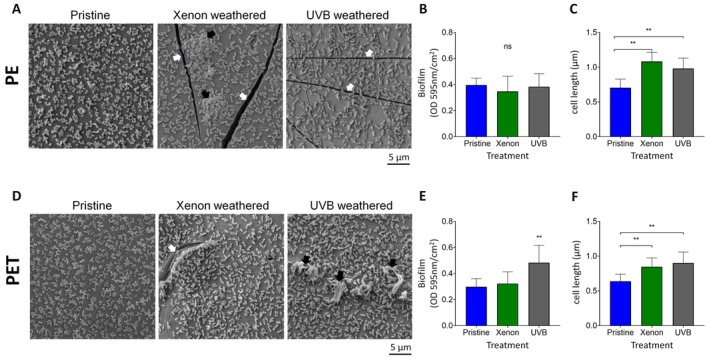
*A dieselolei* colonisation on pristine and photo‐weathered PE and PET. (A, D) SEM images of the colonised *A. dieselolei* bacteria on pristine and photo‐weathered PE and PET surfaces. White arrows‐cracks and micro‐holes on the surface, black arrows‐bacterial biofilm with EPS. (B, E) Crystal Violet quantification of *A. dieselolei* on pristine and photo‐weathered surfaces. **p < 0.01 (one‐way ANOVA, *n* = 8). (C, F) Bacteria length on pristine and photo‐weathered surfaces. **p < 0.01 (One‐Way ANOVA, *n* = 25).

Bacterial cell coverage was evaluated with *A. dieselolei* on both pristine and treated‐PE and PET sheets at the 48‐h incubation time point (Figures 3 and S4). SEM imaging revealed patches of surface‐attached bacteria partially covered with EPS on Xenon‐weathered PE surfaces, but not on the other PE surfaces (Figure 3A). Nevertheless, the Crystal Violet staining values remained similar across treated and pristine PE surfaces (Figure 3B). On the other hand, the UVB weathering treatment increased *Alcanivorax* coverage by approximately 1.6 compared with pristine PET (Figure 3E). The UVB‐weathered PET bacterial cover also included EPS, which may have contributed to the increase in the Crystal Violet staining values (Figure 3D).

We have further noticed an increase in bacterial length on both weathered PE and PET surfaces compared to their pristine counterparts (Figure 3C,F). The average bacterial length on pristine PE was 0.7 ± 0.1 μm compared with 1.0 ± 0.1 μm on Xenon‐weathered PE and 0.9 ± 0.1 μm on UVB‐weathered PE (43% and 29% increase, respectively). Similarly, for PET surfaces, the average bacterial length was 0.6 ± 0.1 μm on pristine samples compared with 0.8 ± 0.1 μm on Xenon‐weathered samples and 0.9 ± 0.1 μm on UVB‐weathered samples (33% and 50% increase, respectively). In all cases, surface‐attached *Alcanivorax* cells were significantly shorter in length compared to free‐living counterparts with an average bacterial length of 1.5 ± 0.4 μm (Figure S5).

### 3.3 Flagella and Swarming Motility in the Different *Alcanivorax* Species

**FIGURE 4:**
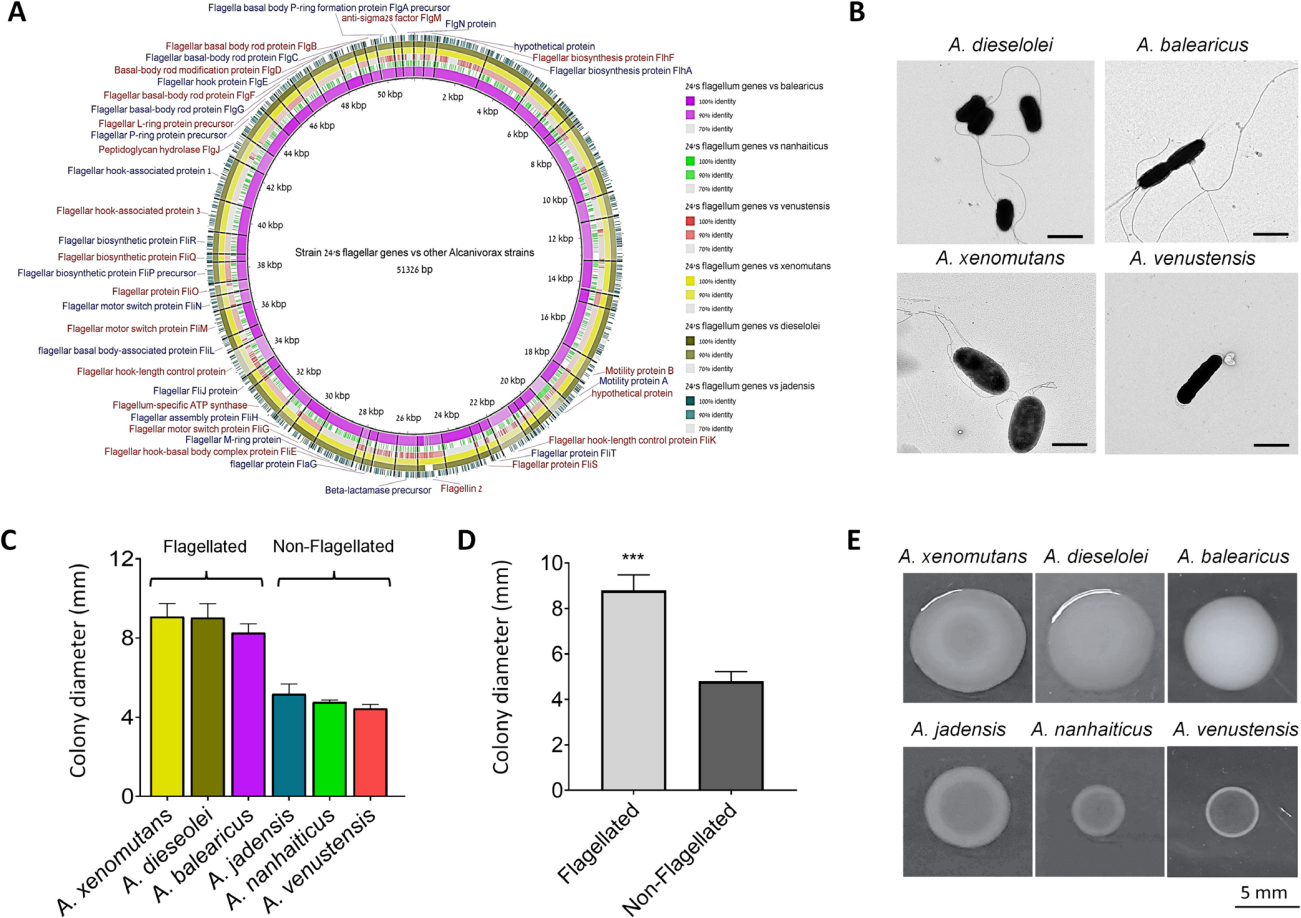
Flagellum presence and swarming motility of the six *Alcanivorax* species. (A) Genetic comparison of flagellar genes between *Alcanivorax 24'*s genome (accession: SNUA00000000) versus the six *Alcanivorax* species. The chart was created using BRIG (version 0.95‐dev.0004). (B) TEM of flagellated *A. dieselolei* , *A. balearicus* , *A. xenomutans* bacteria and a non‐flagellated *A. venustensis* bacterium. Samples were prepared using counterstaining technique (see methods section). (C) Swarming motility of the six *Alcanivorax* species on a soft (0.5%) MB agar 48 h post inoculation. 3 μL droplets of logarithmic stage (OD = 0.3) bacteria were introduced to the agar. Motility was measured by the changes in colony diameter (*n* = 5) of the different species. Species colours correlate to the colours of the BRIG chart. (D) Differences in colony diameter between flagellated and non‐flagellated *Alcanivorax* species. *T* test, ***p < 0.001. (E) *Alcanivorax* colonies' size and morphology 48 h post incubation.

To validate the presence of flagellar genes among *Alcanivorax* species, we compared the *Alcanivorax* sp. *24* flagellum locus (which was previously identified (Zadjelovic et al. 2020a)) to the genomes of the six *Alcanivorax* species that were studied. The BLAST analysis revealed similarity of above 90% in nucleotide sequence of 33 core flagellar machinery genes (e.g., *flaG*) between *A*. sp. *24* and those of *A. balearicus* , *A. dieselolei* and *A. xenomutans*. In contrast, the other *Alcanivorax* species showed either low sequence identity ( *A. nanhaiticus* ), or complete absence of the genes (*A. jadendensis* and *A. venustensis* ), suggesting the absence of a functional flagellum machinery. The genome sizes of the six *Alcanivorax* species varied accordingly, where the flagellated species had a generally larger genome compared to the non‐flagellated ones (Table 1). In agreement with the genomic analyses, phenotypically, a single flagellum was identified using TEM in *A. balearicus* , *A. dieselolei* and *A. xenomutans* species and was not observed in the three other species (Figure 4B).

**TABLE 1:** *Alcanivorax* genome sizes.

Species	Flagellum presence	Genome assembly	No. of contigs	Genome size (Mb)
*Alcanivorax jadensis* T9	−	ASM75665v1	30	3.6
*Alcanivorax venustensis* ISO4	−	ASM1535685v1	91	3.5
*Alcanivorax nanhaiticus* 19‐m‐6	−	ASM75666v1	35	4.1
*Alcanivorax dieselolei* B5	+	ASM30000v1	1	4.9
*Alcanivorax balearicus* MACL04	+	ASM2553214v1	77	4.7
*Alcanivorax xenomutans* JC109	+	2,740,891,819	50	4.4

We further tested the swarming motility of the six *Alcanivorax* species. The flagellated *Alcanivorax* species; *xenomutans*, *dieselolei* and *balearicus*, showed considerable swarming motility, with end‐point colony diameters of 9.07 ± 0.6, 9.02 ± 0.7 and 8.2 ± 0.4 mm, respectively. In contrast, the non‐flagellated species; *jadensis*, *nanhaiticus* and *venustensis* displayed relatively limited motility with colony diameters of 5.17 ± 0.5, 4.7 ± 0.1 and 4.4 ± 0.2 mm, respectively (Figure 4C). Overall, the swarming motility was significantly higher in the flagellated versus non‐flagellated *Alcanivorax* species groups (Figure 4D). The colony morphology also differentiated between flagellated and non‐flagellated species, with the non‐flagellated species exhibiting colonies with a denser outer ring, and the flagellated ones exhibited colonies with either inner denser ring (*A. xenomutans*) or unified density throughout the colony (*A. dieselolei* and *A. balearicus*; Figure 4E).

### 3.4 The Effect of Flagellum Disturbance on *Alcanivorax* Colonisation

**FIGURE 5:**
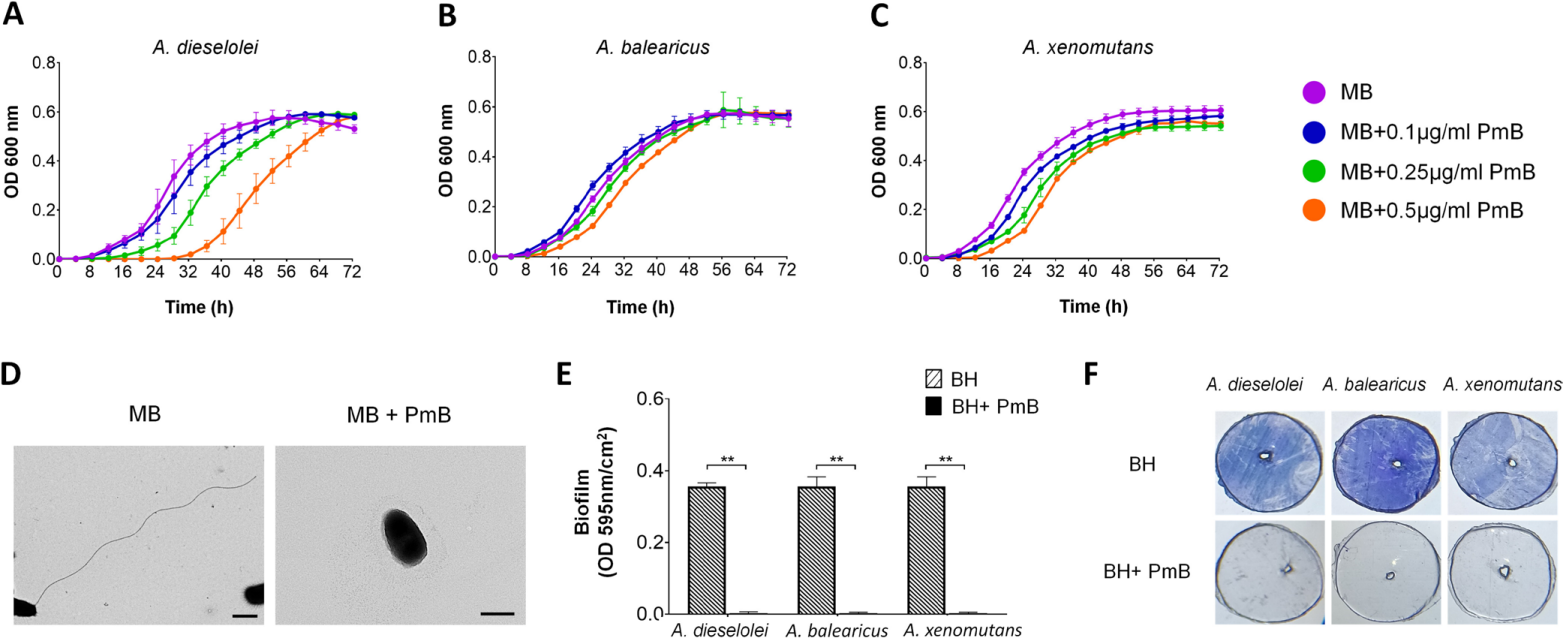
Growth, motility and biofilm formation of *Alcanivorax* species in the presence of sub‐inhibitory doses of PmB. (A–C) 72 h growth curves (OD 600 nm) of *A. dieselolei* , *A. balearicus* and *A. Xenomutans* in MB and MB supplemented with 0.1, 0.25 and 0.5 μg/mL PmB. (D) *A. dieselolei* in MB medium with full‐length flagellum (left) versus *A. dieselolei* in MB medium containing 0.1 μg/mL PmB, with no flagellum (right). Scale: 1 μm. (E, F) Colonisation inhibition of flagellated *Alcanivorax* species by 0.1 μg/mL PmB. Colonisation experiments were conducted in BH media. Bacterial biofilms were assessed using Crystal Violet staining 48 h post‐inoculation of *A. dieselolei* , *A. balearicus* and *A. Xenomutans* (E). Stained and unstained surfaces are presented in (F) *p < 0.01, **p < 0.001 (two‐way ANOVA).

To test the hypothesis that the flagellum presence is essential to bacterial adhesion to plastic in the marine environment, we used sub‐concentrations of PmB as a flagellar inhibitor (Giacomucci et al. 2019). To determine the specific PmB sub‐concentration for the rapid colorizer *Alcanivorax* species, we evaluated the effect of different sub‐concentrations of PmB on bacterial viability (Figure 5A–C). *Alcanivorax* bacteria cells remained viable across all sub‐concentrations used and achieved similar culture growth values after 72 h. However, at PmB concentrations of 0.2 and 0.5 μg/mL, the growth rates were slightly slower, particularly in *A. dieselolei* culture, which showed an ~18‐h longer lag phase in the 0.5 μg/mL concentration. Following the above results, PmB 0.1 μg/mL concentration was selected for subsequent experiments.

The disturbance of flagella with a sub‐inhibitory dose of 0.1 μg/mL PmB was confirmed by TEM imaging. The PmB‐treated *A. dieselolei* cells did not show a flagellum at all (Figure 4D). Similar results were observed in *A. balearicus* and *A. xenomutans* (data not shown) that either lacked a flagellum or exhibited incomplete or fragmented ones.

We further assessed the swarming motility of the flagellated *Alcanivorax* species on soft agar in the presence of 0.1 μg/mL PmB. The results showed a significant reduction in swarming motility, with 21%, 10% and 4% reduction in the end‐point colony diameter of *A. balearicus* , *A. dieselolei* and *A. xenomutans*, respectively (Figure S6).

To test the effect of flagellum disturbance on colonisation, we performed PE colonisation assays in the presence of 0.1 μg/mL PmB, as described previously. The Crystal Violet staining results indicated a complete inhibition of colonisation for all three flagellated species in response to the treatment, suggesting that the flagellum is essential to the mechanism of rapid plastic surface colonisation in *Alcanivorax*.

## 4 Discussion

The results of this study show that the *Alcanivorax* genus exhibits selective colonisation patterns on plastic surfaces, with polymer type and weathering state significantly influencing initial bacterial adhesion and/or morphology. Polyolefin plastics (PE and PP) supported higher colonisation rates compared to PET, as demonstrated by Crystal Violet staining and SEM imaging. Among the six tested *Alcanivorax* species, only three ( *A. balearicus* , *A. dieselolei* and *A. xenomutans*) rapidly colonised plastics, correlating with their phylogenetic grouping and the presence of functional flagella. We confirmed that the presence of flagella is critical for colonisation, as flagellar disturbance with sub‐inhibitory doses of polymyxin B antibiotic completely blocked bacterial adhesion to the plastic surfaces.

A higher initial colonisation rate of *Alcanivorax* on polyolefins compared to PET surfaces was demonstrated here, as well as in previous field and aquarium experiments (Marsay et al. 2022). This preferential colonisation correlates with the ability of *Alcanivorax* to degrade polyolefins. The polyolefin plastics, including PE and PP, similar to other linear‐chain alkanes, are essentially matrixes of saturated hydrocarbon molecules that vary in their length rather than in their basic chemical structure. The hydrophobic nature of *Alcanivorax*, which was studied in the context of adhesion to hydrocarbon oil droplets, likely facilitates their interaction with non‐polar surfaces through hydrophobic interactions. For example, the production of glycine‐glycolipids on the cell surface of *A. borkumensis* SK2 enhances hydrophobicity, improving adhesion to hydrophobic surfaces (Karmainski et al. 2023). Furthermore, it was recently shown that *Alcanivorax* hydrophobicity may be increased following 5 days of pre‐exposure to linear hydrocarbons (Prasad et al. 2023). Similar strategies to increase hydrophobicity for enhancing bioavailability have been observed in other bacteria, such as *Rhodococcus erythropolis* (de Carvalho et al. 2009) and *Mycobacterium* sp. LB501T (Wick et al. 2002). Given the relatively low abundance of *Alcanivorax* bacteria in the water column of less than 1% (Davidov et al. 2020) and its relatively slow generation time (~4–8 h, species‐depended), we wondered how *Alcanivorax* can colonise plastic surfaces so rapidly and become one of the most dominant bacterial genera within the initial colonisation period after the introduction of a new plastic surface to the marine environment (Latva et al. 2022).

We first tested whether pre‐existing, non‐biological plastic photo weathering may be a factor in supporting rapid surface *Alcanivorax* attachment. Using FTIR, we detected chemical alterations in the form of newly formed carbonyl groups in Xenon‐ and UVB‐treated PE surfaces; however, we could not detect any FTIR spectrum changes in the equivalent PET surfaces. On the other hand, both PE and PET surfaces showed physical alterations as well as a decrease in hydrophobicity (and an increase in polarity) following photo‐weathering treatment. While three of the six *Alcanivorax* species rapidly colonised the hydrophobic pristine polyolefins and PET surfaces, we noticed enhanced colonisation of *A. dieselolei* on the UVB‐treated PET and a significant increase in bacterial cell length on both Xenon‐and UVB‐weathered PE and PET compared to their pristine counterparts. The increase in *A. dieselolei* cell length may reflect the better compatibility of cell surface polarity to the more polar plastic surfaces that underwent photo‐weathering treatment (Zheng et al. 2021). Furthermore, sporadic EPS patches were recorded only on treated surfaces, suggesting that EPS secretion by *A. dieselolei* is induced following photo weathering, which again may be related to increased surface polarity.

The second factor, which was tested in the context of *Alcanivorax* colonisation of plastic, was the presence of a flagellum. We have shown that the flagellated species, *A. dieselolei* , *A. balearicus* and *A*. *xenomutans*, rapidly adhere to and colonise all the tested plastic surfaces, while the three non‐flagellated species, *A. jadensis* , *A. nanhaiticus* and *A. venustensis* , did not. Flagella are important for bacterial motility and chemotaxis, enabling bacteria to navigate toward suitable substrates (Colin et al. 2021; Vilas Boas et al. 2024; Hershey 2021). Moreover, flagella and flagellum‐based motility play a critical role in adhesion to surfaces in some bacteria (Friedlander et al. 2015; O'Toole and Kolter 1998). More specifically, it was demonstrated that the flagellum is essential for the adhesion of *E. coli* to plastic surfaces (Zhang et al. 2023). By disrupting the flagellum function using a chemical inhibitor, PmB, we demonstrated for the first time its necessity for adhesion of *Alcanivorax* bacteria on different plastic surfaces. Nevertheless, it is yet to be discovered what necessary functions are provided by the flagellum for the rapid adhesion of *Alcanivorax* to the plastic surfaces. While the flagellum is used for chemotactic motility, its presence in bacteria does not necessarily indicate chemotaxis (Slightom and Buchan 2009). On the other hand, a linear alkane‐based chemotaxis mechanism was proposed for *A. dieselolei* (Wang and Shao 2014) and may also play a role in chemotaxis to polyolefin surfaces. The alternative is that the *Alcanivorax* flagellum provides undirected motility, therefore promoting random encounters with the surface rather than directed ones. While our results demonstrate that the absence of a functional flagellum significantly reduces the in vitro swarming motility in a soft agar setup, it is far from completely abolishing the bacterial cell motility. Given the above, we hypothesise that the function of the *Alcanivorax* flagellum in the context of plastic surface adhesion is also to promote physical adhesion to the surface. However, we note that this hypothesis needs to be further explored. Moreover, further study about the presence and/or formation of fimbria, pili, EPS and quorum sensing is important for the understanding of the following steps in *Alcanivorax* colonisation and biofilm formation.

Our findings emphasise the importance of polymer environmental weathering, flagellum and flagellum‐based motility in the initial adhesion of certain *Alcanivorax* bacteria to plastic polymers, specifically polyolefins. The initial adhesion is an essential step for the possible utilisation of the plastic as a carbon and energy source and for the subsequent downstream processes that lead to the establishment of a fully developed plastisphere community.

## Author Contributions

**Keren Davidov:** writing – original draft, methodology, conceptualization, visualization, investigation, software, formal analysis, project administration, writing – review and editing, validation. **Sheli Itzahri:** formal analysis, methodology, writing – review and editing, investigation. **Aiswarya Kartha:** methodology, formal analysis, writing – review and editing, investigation. **Gilad Orr:** formal analysis, data curation, writing – review and editing, methodology. **Ziv Lang:** software, writing – review and editing, methodology. **Shiri Navon‐Venezia:** conceptualization, writing – review and editing, funding acquisition. **Matan Oren:** writing – review and editing, conceptualization, visualization, supervision, resources, project administration, writing – original draft, methodology, funding acquisition.

## Conflicts of Interest

The authors declare no conflicts of interest.

## Supporting information


Data S1.

## Data Availability

Data sharing not applicable to this article as no datasets were generated or analysed during the current study.
