# Supplementary material for: Rapid Colonisation of Plastic Surfaces by Marine Alcanivorax Bacteria Is Flagellum‐Dependent and Influenced by Polymer Type and Photo‐Weathering State
Source: Environ Microbiol. 2025 May 2;27(5):e70102. doi: 10.1111/1462-2920.70102 (PMC12046545; doi:10.1111/1462-2920.70102)
Supplement: Supplementary file 1 — Data S1. [file EMI-27-e70102-s001.docx]

**Rapid Colonization of Plastic Surfaces by Marine *Alcanivorax* Bacteria is Flagellum-dependent and Influenced by Polymer type and Photo-Weathering State**

**Supplementary information**

Keren Davidov^1^, Sheli Itzahri^1^, Aiswarya Kartha^1^, Gilad Orr^2^, Ziv Lang^1^, Shiri Navon-Venezia^1,3^ & Matan Oren^1^*

^1^ Department of Molecular Biology, Ariel University, Ariel, Israel.

^2^ Physics Department, Crystal Physics Laboratory, Ariel University, Ariel, Israel.

^3^ The Sheldon Adelson School of Medicine, Ariel University, Ariel, Israel.

*matanor@ariel.ac.il


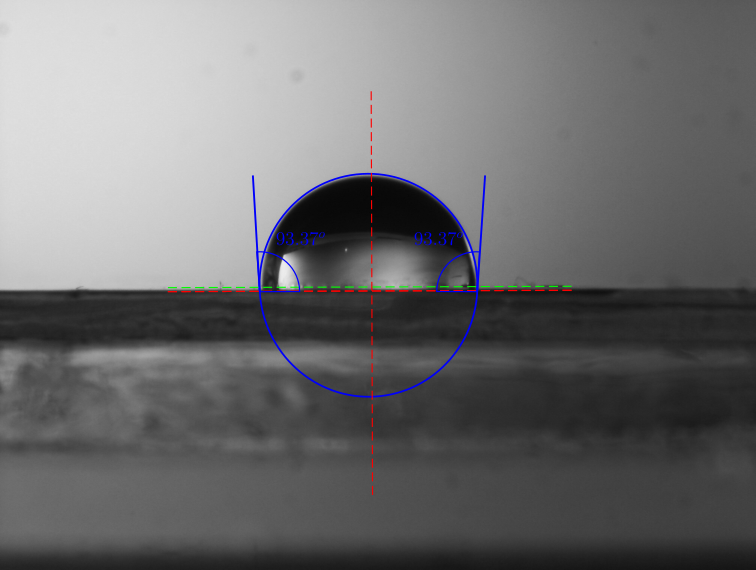


**Supplementary Figure S1.** **Sessile drop test.** Image analysis for the PET that was photo-weathered by UVB for 7 days.


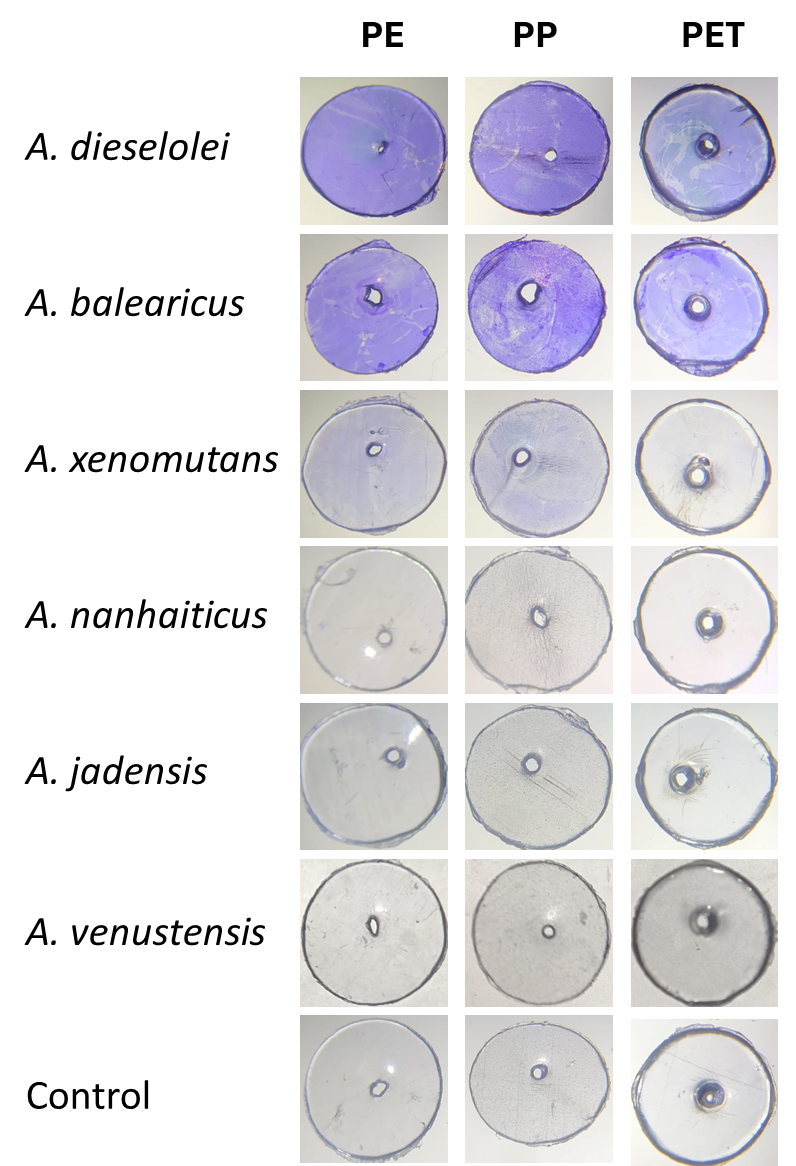


**Supplementary Figure S2. Colonization of the six Alcanivorax strains on plastic surfaces.** Biofilm formation on PE, PP, and PET surfaces visualized with Crystal Violet staining after 48 hours.


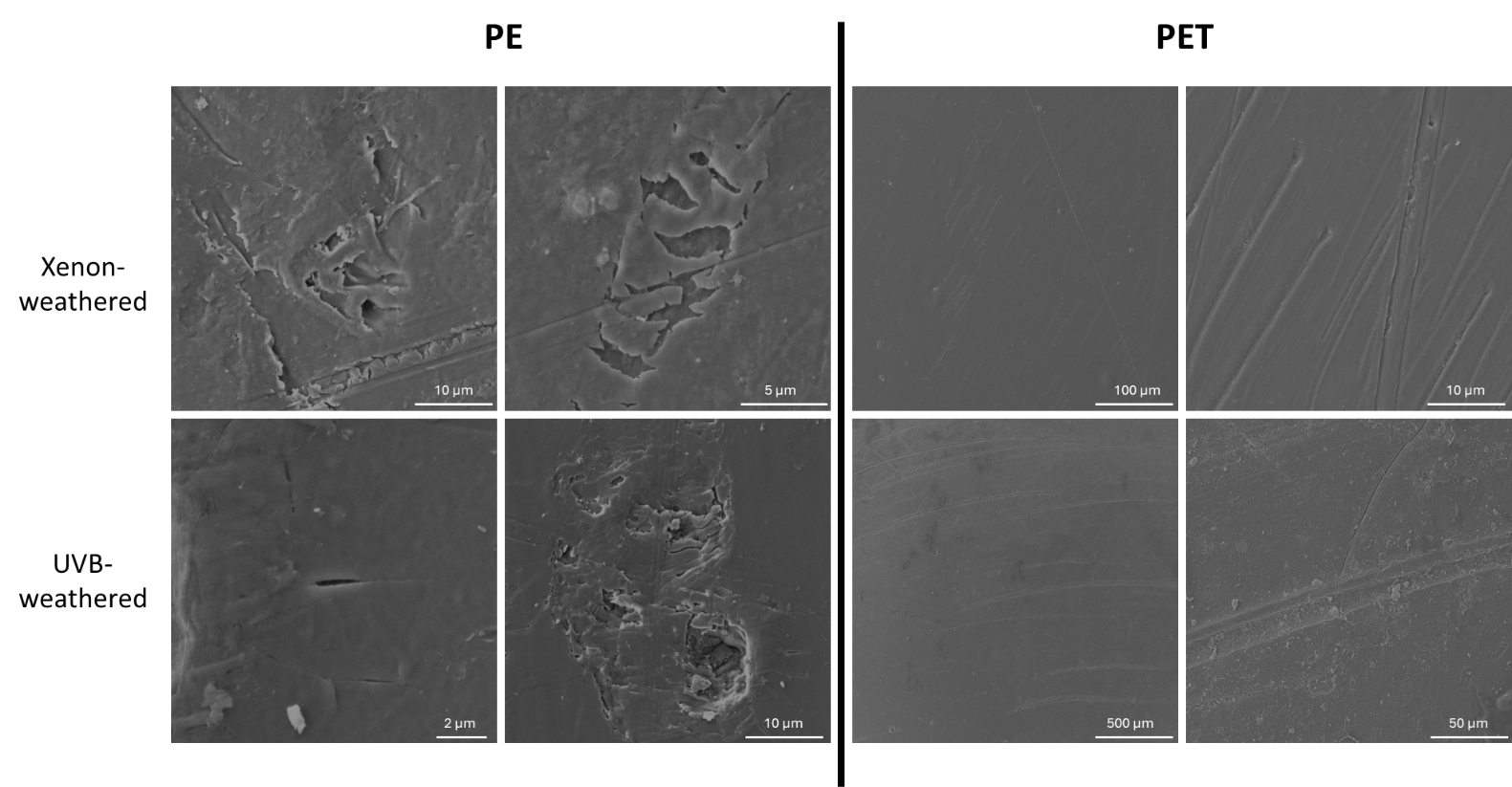


**Supplementary Figure S3. SEM images of PE and PET surfaces after exposure to artificial daylight (Xenon) and ultraviolet radiation (UVB light source).**


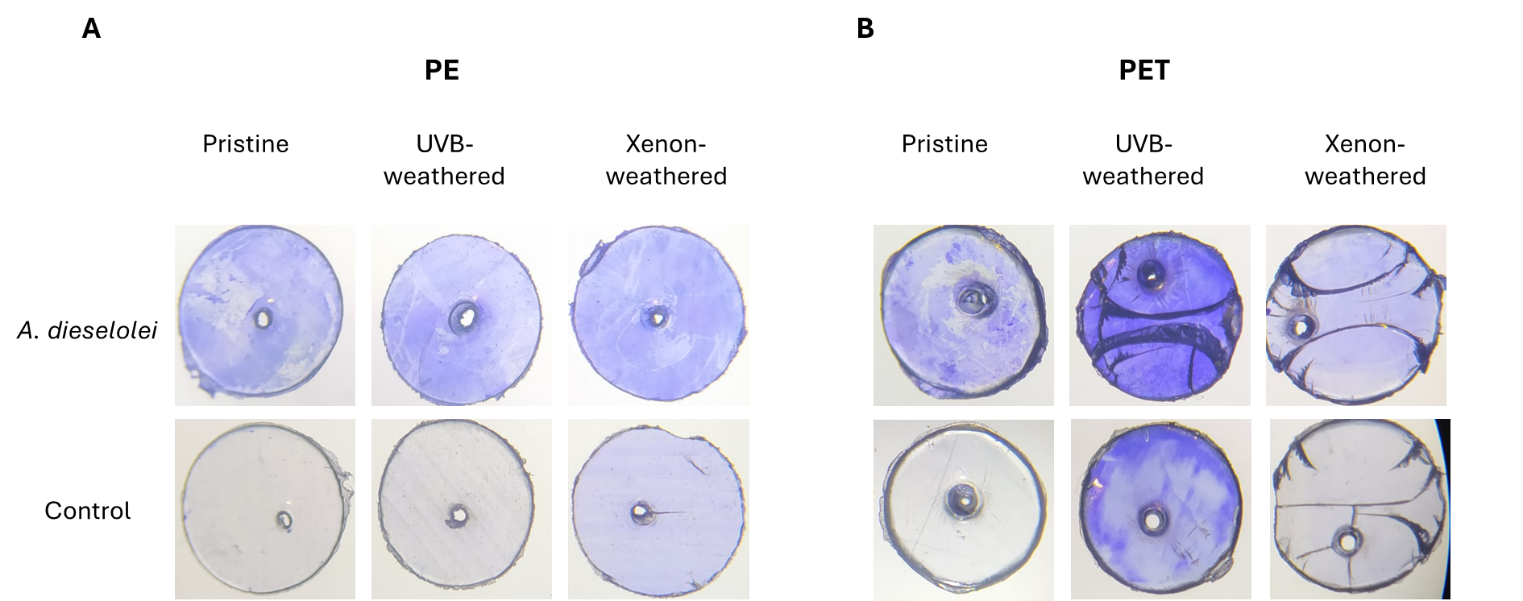


**Supplementary Figure S4. A. dieselolei colonization on pristine and photo-weathered PE and PET.** Surfaces visualized with Crystal Violet staining after 48 hours. (A) PE surfaces. (B) PET surfaces.


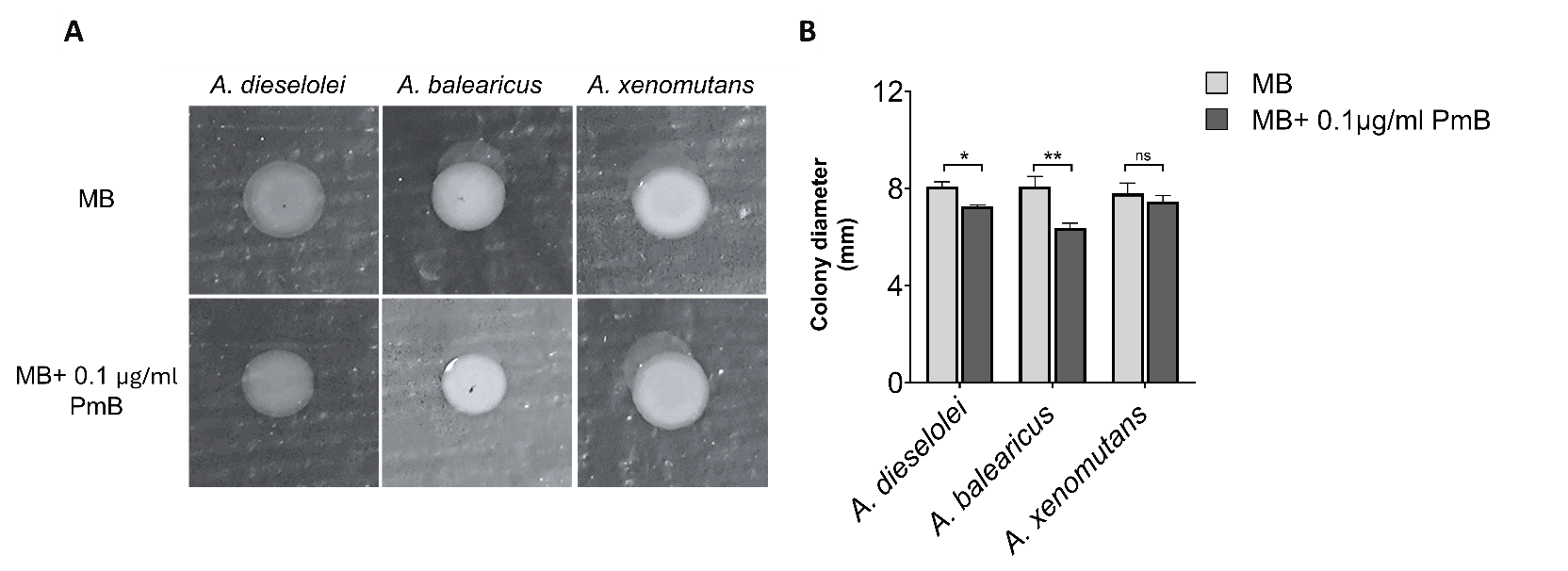


**Supplementary Figure S6. Inhibition of Alcanivorax swarming motility by sub-inhibitory concentrations of PmB.** (A) Images of Alcanivorax colonies on MB soft agar (0.5%) plates with 0.1 µg/ml PmB after 30h. (B) Colony diameter between MB soft agar with and without PmB treatment. *p < 0.05, ** p < 0.01 (Two-Way ANOVA), n=5.


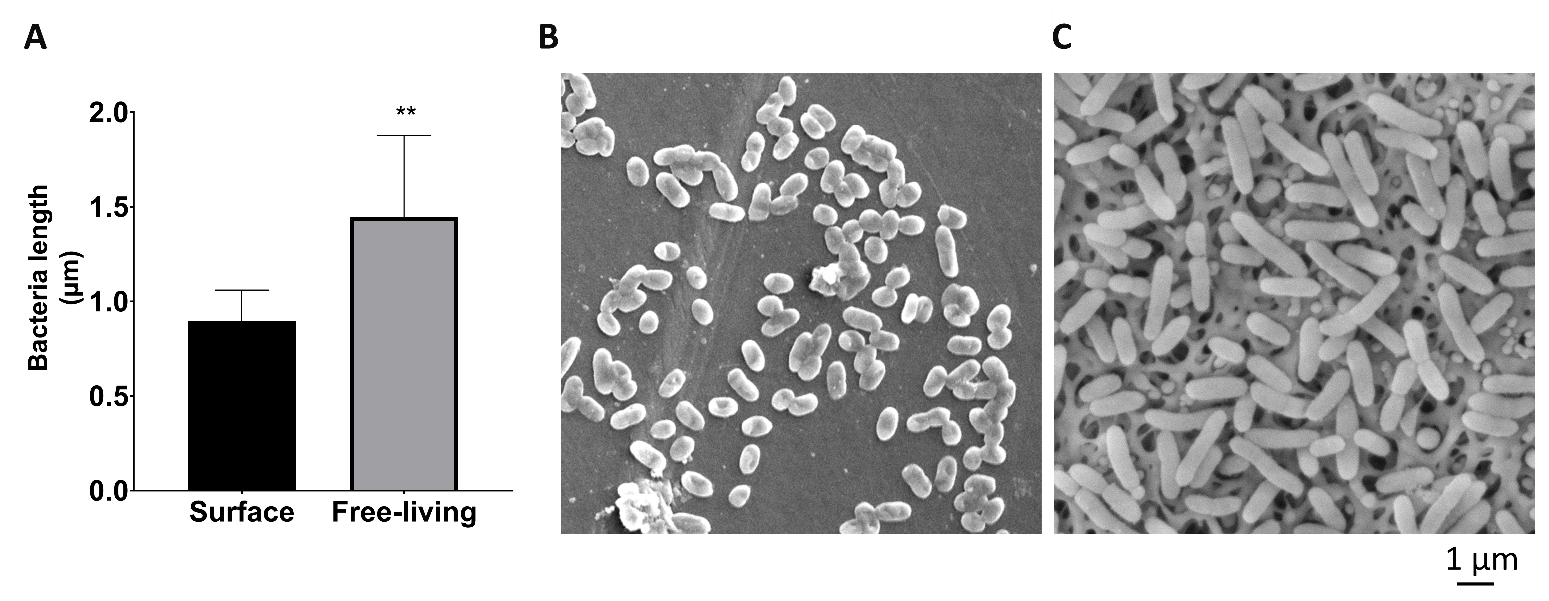


**Supplementary Figure S5. A. dieselolei cell morphology on PE surfaces and free-living conditions.** (A) Bacterial cell length on PE surfaces vs. free-living cells after 24 hours in MB media. (B, C) SEM images of A. dieselolei bacteria on PE surfaces (B) and free-living cells on 0.22 µm filter (C). Statistical analysis was performed with t-test; ** p < 0.001.
